# ProPIP: a tool for progressive multiple sequence alignment with Poisson Indel Process

**DOI:** 10.1186/s12859-021-04442-8

**Published:** 2021-10-24

**Authors:** Massimo Maiolo, Lorenzo Gatti, Diego Frei, Tiziano Leidi, Manuel Gil, Maria Anisimova

**Affiliations:** 1 Institute of Applied Simulation, School of Life Sciences and Facility Management, Zurich University of Applied Sciences (ZHAW), Schloss 1, Postfach, 8820 Wädenswil, Switzerland; 2 Swiss Institute of Bioinformatics (SIB), Quartier Sorge - Batiment Amphipole, 1015 Lausanne, Switzerland; 3 Institute of Information Systems and Networking, University of Applied Sciences and Arts of Southern Switzerland, Galleria 2, Via Cantonale 2c, 6928 Manno, Switzerland

**Keywords:** Indel evolution, Dynamic programming, Poisson Indel Process, Multiple sequence alignmnet, Evolutionary alignment, Alignment software

## Abstract

**Background:**

Current alignment tools typically lack an explicit model of indel evolution, leading to artificially short inferred alignments (i.e., over-alignment) due to inconsistencies between the indel history and the phylogeny relating the input sequences.

**Results:**

We present a new progressive multiple sequence alignment tool ProPIP. The process of insertions and deletions is described using an explicit evolutionary model—the Poisson Indel Process or PIP. The method is based on dynamic programming and is implemented in a frequentist framework. The source code can be compiled on Linux, macOS and Microsoft Windows platforms. The algorithm is implemented in C++ as standalone program. The source code is freely available on GitHub at https://github.com/acg-team/ProPIP and is distributed under the terms of the GNU GPL v3 license.

**Conclusions:**

The use of an explicit indel evolution model allows to avoid over-alignment, to infer gaps in a phylogenetically consistent way and to make inferences about the rates of insertions and deletions. Instead of the arbitrary gap penalties, the parameters used by ProPIP are the insertion and deletion rates, which have biological interpretation and are contextualized in a probabilistic environment. As a result, indel rate settings may be optimised in order to infer phylogenetically meaningful gap patterns.

## Background

Multiple sequence alignment (MSA) is a fundamental task required by most genomic analyses, with a multitude of alignment tools already available. Due to the inherent computational complexity of MSA inference, several heuristics have been proposed. The progressive approach is one of popular strategies that involves aligning pairs of sequences or alignments from the tips towards the root along the tree structure that represents the evolutionary relationship of the input sequences (i.e., the tree leaves). At each internal node a dynamic programming instance (DP) aligns the partial solutions present in its two child nodes. In general, the partial MSA solutions at each inner node and hence also the final MSA at the tree root correspond to the local optima obtained by maximizing the partial solution in a smaller space spanned by local pairwise alignments. This approximation is however widely accepted and represents the status quo today. Typically the DP algorithm scales quadratically with the average length of the sequences [1, 2].

Classically, however, aligners only consider substitutions and the length distribution of the observed sequence gaps. These methods typically do not explicitly model the evolution of indels (insertions/deletions). This shortcoming can lead to a disconnect between the history of indel events and the phylogenetic relationship of the sequences, and the consequent visible distortions are over-alignment (*i.e*., artificially short alignments). Among notable exceptions are PRANK [3] and PrographMSA [4], but both account for indel evolution algorithmically rather than using an explicit mathematical model. The inclusion of more complicated scenarios requires that the underlying evolutionary model is more sophisticated, which almost always goes along with a greater computational complexity. A typical example of an explicit indel model that represented a paradigm shift was the TKF91 model [5] whose calculation of the marginal likelihood requires an exponential time in the number of sequences, or the more recent Poisson Indel Process PIP [6] that reduced the complexity to linear. Whilst TKF91 and PIP are mathematically very different, both models explicitly describe indel evolution directly on a phylogeny.

The PIP model has been proposed as a new evolutionary model together with formulas to efficiently calculate the marginal likelihood given unaligned sequences, the evolutionary parameters and a tree that relates the input sequences. This makes it possible to measure the goodness of fit of this model to a pool of candidate MSAs given the model parameter. The candidate with the highest optimised log-likelihood is considered to be the best description of the unaligned data under a fixed model. Recently we developed a progressive MSA inference method that generates MSA candidates and scores them under the PIP model [7]. It was shown that this method, therefore, infers gaps in a phylogenetically consistent and meaningful way. In addition, the use of an explicit indel model allows to make inferences about the rates of insertions and deletions, replacing the need for gap penalty parameters, which are known to be difficult to set and interpret.

## Implementation

Here we present the ProPIP software, which implements our originally published progressive MSA inference method based on PIP [7], and also introduces new features, such as stochastic backtracking and parallelisation (as described below). According to the PIP model, insertions are Poissonian events on a phylogeny that add single characters to a sequence. Once inserted, a character evolves via a continuous time Markov process of substitutions and deletions along the phylogeny relating the sequences. The intensity of insertions and deletions is parameterized by two rates that determine the type of homology and consequently the gap pattern in the final alignment. By modifying these parameters different homology hypotheses can be compared in a model-based framework. Thus, instead of the traditional gap penalties (which are typically set arbitrarily), the parameters used by ProPIP are the insertion and deletion rates, which have biological interpretation and are contextualized in a probabilistic environment.

**Fig. 1:**
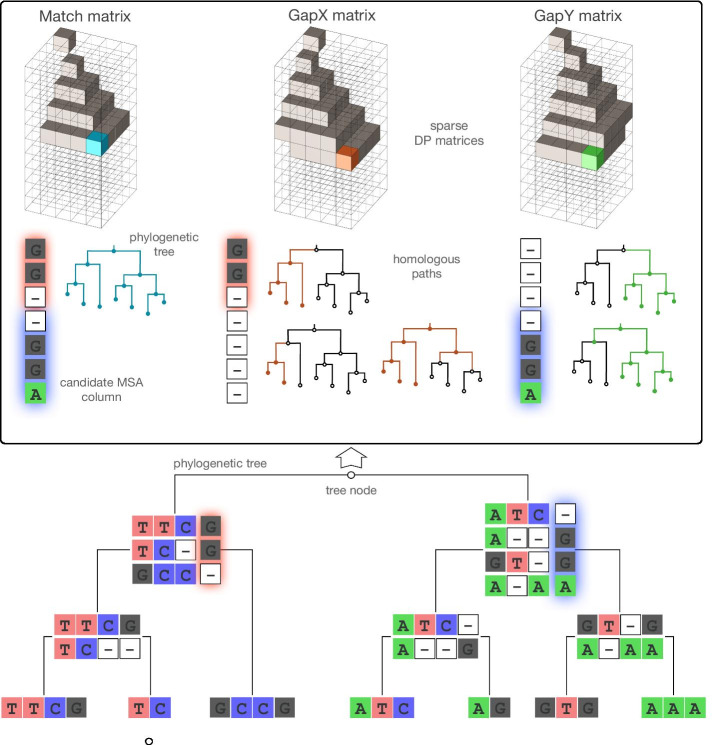
Algorithm scheme. At each internal node of the guide tree an instance of the algorithm aligns the homology paths of the two sub-alignments present in the children nodes. Each instance of the aligner requires 3 sparse three-dimensional DP matrices for Match, GapX and GapY (shown in the figure). These matrices contain the column by column likelihood of the 3 respective states. The state, among the 3 possible choices, with the highest likelihood is saved in a fourth traceback matrix (not shown). At the end, the final MSA is generated by traversing the traceback matrix backwards following the path that generates the highest likelihood at each step. GapX and GapY states require a marginalization of multiple possible homology paths. Some of these are highlighted in the figure. The existence of a character is illustrated by the coloured strokes on the trees

ProPIP can align both nucleotide and protein sequences. The overall complexity of our progressive algorithm is $\mathcal {O}(Nl^3)$, for *N* taxa and an average input sequence length *l*. Further running time reductions are possible. For example, recently we proposed a strategy to accelerate alignment inference by trimming the original DP matrix [8]. The method is implemented in the frequentist framework, where log-likelihood scores under PIP are used as an optimality criterion. In a progressive fashion, ProPIP traverses a guide tree phylogeny from the leaves towards the root according to one of the two different modes: (1) using the Dynamic Programming (DP), and (2) using the Stochastic Backtracking version (see also Fig. 1. These are briefly described below.

### Dynamic programming

ProPIP proceeds progressively from leaves towards the root of a guide tree. By default, at each internal node the algorithm aligns the evolutionary histories in the left and right subtrees by full maximum likelihood (ML) using DP, to obtain the homology history at the current node. More specifically, at each node the likelihood computation marginalizes over all possible indel and substitution scenarios given the sub-alignments obtained for the child nodes in the previous steps of the progressive algorithm. This includes homology histories where all characters have been deleted, i.e. unobserved or “empty” columns. In a given node, any two MSA columns from the child nodes can be aligned in three ways: either they matched, or any of the two columns is aligned with a column full of gaps. Each of these three states can in turn imply a number of scenarios which, depending on the depth of the tree and the number of gaps present, can also be large. The algorithm computes the likelihood for each of the three scenarios, In particular, we consider all possible places where a character may have been inserted along the phylogeny and all possible points where it may have been deleted. All these homology paths are listed and marginalised into a single likelihood value, without having to make a choice of one scenario (e.g. based on parsimony or ML).

Our DP is locally optimal, i.e. in each internal node the two sub-alignments are aligned by full ML. Progressive application of DP, however, does not lead to a globally optimal solution. To overcome this greedy behaviour, we have enhanced our method with SB - stochastic backtracking [9], adapted to the PIP model.

### Stochastic backtracking

SB provides an ensemble of sub-optimal candidate solutions, distributed according to their individual probabilities. During progressive alignment with the SB option, SB is applied at each internal node. Instead of aligning only the optimal histories at the two children, the aligner generates an ensemble (e.g. alignment.sb_solutions=4) of histories combining samples from the distributions at the children nodes. Therefore, the SB version of the algorithm reduces the chances to be trapped in local optima produced by the greedy nature of the default progressive DP.

SB is parameterised by a temperature *T* (e.g. alignment.sb_temperature=0.8), which tunes the deviation from the optimal alignment. For $T = 0$ SB returns the optimal alignment, falling back to classical DP. By setting $T \rightarrow \infty$, each alignment becomes equiprobable and the solution is therefore random. In the range $0< T < \infty$ the parameter controls the deviation from the optimal alignment allowing, gradually, the generation of sub-optimal alignments.

### Substitution models

ProPIP can align either nucleotide (alphabet=DNA) or amino acid (alphabet=Protein) sequences, based on different substitution models available in the Bio++ library [10]. Among these are the nucleotide models are JC69, K80, HKY85, and GTR, and the amino acid models JTT, WAG, and LG. All models are extended with PIP. For a complete list of the substitution models available see the Bio++ documentation.

In addition, users can choose to account for Across-Site Rate Variation (ASRV), which is implemented as a discretised $\Gamma$ distribution (default), or alternatively as exponential or Gaussian distributions, with user-defined number of discrete categories.

### Initial tree and indel rate inference

Providing a reasonable initial guide tree [11] and indel rates helps to make the MSA inference more accurate. These can be provided by the user when known. If the guide tree is not provided then ProPIP first computes a distance matrix from the pairwise alignments which is then used to infer a guide tree as a rooted BioNJ tree [10, 12].

The same applies to indel rates (insertion rate and deletion rate), which are inferred from the data when not provided by the user. We compute the initial indel rate values of the PIP model from pairwise alignments using the Needleman-Wunsch algorithm with gap opening and extension penalties for nucleotide sequences and a Grantham distance-based scoring method for amino acids [13]. The indel rates are calculated from the pairwise alignments as follows. The phylogeny and indel rate parameter values imply expectations on the number of gap/non-gap states (or gap patterns) in alignments. Each position in a pairwise alignment belongs to one of three possible patterns: either no gap is present, or a gap is present in one of the two sequences. Given that we need to estimate two parameters ($\lambda$ and $\mu$) this leads to an overdetermined system of equations. We solve this system for each pairwise alignment using a non-linear least-squares algorithm [14, 15]. Then we take an average over all estimates to obtain the indel rates for the progressive alignment.

Finally, the various indel rates are averaged to obtain the initial insertion and deletion rate. The estimated indel rates eventually determine the resulting MSA gap pattern.

### Parameter optimization

ProPIP allows the optimisation of model parameters, such as indel rates or the instantaneous substitution rates between characters. These features are inherited from Bio++ libraries. When requesting parameter optimisation, the system automatically instantiates the appropriate OptimizationTools class object. As input, this object receives a pointer to the likelihood function, which can be evaluated under PIP if the user wishes to invoke this evolutionary model. It is also possible to specify the maximum number of iterations or a tolerance value at which the optimisation ends. The user can monitor the optimisation progress and the final values in the two files “profiler” and “messenger”. Among the various Bio++ functions that ProPIP couples with the PIP model are the Brent and BFGS optimisation routines. In both cases the method optimises all parameters until convergence, respecting the requested thresholds or the maximum number of steps. If, on the other hand, the user desires to fix parameters at given values, this can be specified via the “None” optimisation option.

The syntax is the following: optimisation=ND-Brent(derivatives=Brent,nstep=1000). It is also possible to specify which parameters to ignore, for example if the user wants to optimise the insertion rate $\lambda$ and the deletion rate $\mu$ but not $\kappa$ of the K80 substitution model then the following should be specified: optimisation.ignore_parameter = K80.kappa. For more details see the wikipages on our github website and the Bio++ manual.

### Parallelization

To reduce the computational time, ProPIP was parallelised. We use the open source version of Intel Thread Building Blocks library available at https://github.com/oneapi-src/oneTBB, which can be activated by the user (see documentation). The following parallelisation options are provided:

*parallel_for:* In this option, for-loops have been rewritten to exploit tbb::parallel_for loops provided by Intel TBB. This loop instruction allows to split the looping range into smaller chunks that are then executed in parallel by TBB’s tasks. This approach has been applied to the vector and matrix initialization loops and to the actual dynamic programming forward phase, where the likelihood matrices are computed and the maximum likelihood score is sought. To preserve the optimisation algorithms in the parallel execution context, the local optimum comparison and variable update have been protected using a locking mechanism (tbb::mutex). The necessity of this lock clearly influences and limits the achievable parallelism of this approach.

*TBB Task:* The TBB Task optimization provides more flexibility during the parallelization. Instead of parallelizing the internal loops this approach focuses to parallelize each node. The tree topology is processed in a post-order traversal, from the leaves towards the root of the tree. Starting from the root node each node creates 2 tasks (1 for each child node) and executes them in parallel before doing its own processing. This recursive process is repeated until the leafs of the binary tree are reached and the actual execution begins. Each node is executed as a separate parallel task, which leads to a more dynamic parallelism. Table 1 shows the speed-up values for some *n*-taxa trees and as the number of columns to be aligned increases. The speed-up factor improves when increasing the number of taxa while it remains constant when augmenting the number of columns.

**Table 1:** The table shows the computational times as a function of the number of taxa and the number of columns to be aligned

taxa\cols	100	200	400	800
8	0.005; 0.004; 0.003 (1.18; 1.37)	0.049; 0.035; 0.032 (1.40; 1.55)	0.383; 0.270; 0.268 (1.42; 1.43)	2.675; 1.769; 1.842 (1.51; 1.45)
16	0.011; 0.005; 0.005 (2.17; 2.24)	0.120; 0.057; 0.059 (2.11; 2.02)	0.750; 0.338; 0.357 (2.22; 2.10)	5.604; 2.624; 2.630 (2.14; 2.13)
32	0.021; 0.008; 0.014 (2.53; 1.49)	0.149; 0.061; 0.056 (2.46; 2.65)	1.374; 0.456; 0.458 (3.01; 3.00)	10.630; 3.530; 3.929 (3.01; 2.71)
64	0.037; 0.013; 0.012 (2.85; 3.14)	0.407; 0.123; 0.116 (3.31; 3.50)	2.612; 0.743; 0.737 (3.52; 3.54)	22.855; 6.220; 6.925 (3.67; 3.30)
128	0.138; 0.037; 0.030 (3.76; 4.52)	0.966; 0.222; 0.214 (4.36; 4.52)	5.711; 1.282; 1.210 (4.46; 4.72)	49.183; 10.344; 11.060 (4.75; 4.45)
256	0.281; 0.046; 0.051 (6.15; 5.55)	2.064; 0.320; 0.317 (6.45; 6.51)	11.694; 2.171; 1.929 (5.39; 6.06)	98.428; 17.529; 16.762 (5.62; 5.87)

The times are given in minutes for the single core version; TBB Task; parallel_for and in brackets the relative speed-up values

*Thread control:* to have a better control of the parallel execution environment it is possible to limit the number of threads the TBB library will create and use to execute the parallel tasks. Finally, also thread pinning can be enabled, which allows to specify the CPUs the threads will be assigned to.

## Results

**Fig. 2:**
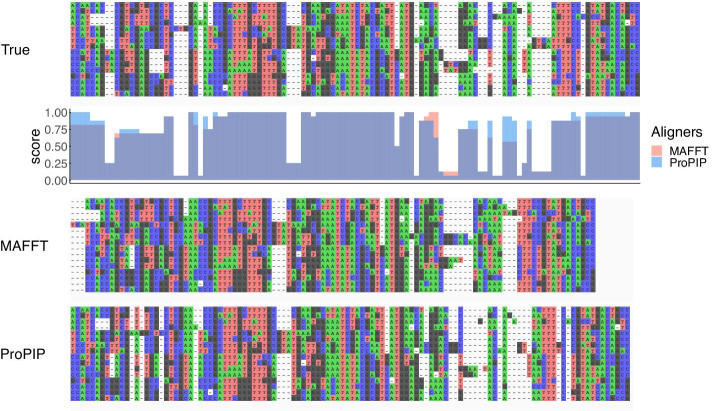
Overalignment example block #1. This figure shows the true MSA (top) obtained as described in section Results, the alignment obtained with MAFFT (middle), and with ProPIP (bottom). The 3 MSAs represent a portion of the entire alignment, namely columns 1–116, 1–107, and 1–114 for true, MAFFT, and ProPIP, respectively. For ProPIP we selected the MSA with the highest likelihood and not the one with the best Q score [20]. It can be noted that MAFFT tends to over-align, while the gap pattern resulting from ProPIP is more compatible with the true MSA. The similarity value (AlignStat v1.3.1 [19]) are $75\%$ for MAFFT and $82\%$ for ProPIP [27]. Column by column similarity values are shown under true MSA for both aligners

**Fig. 3:**
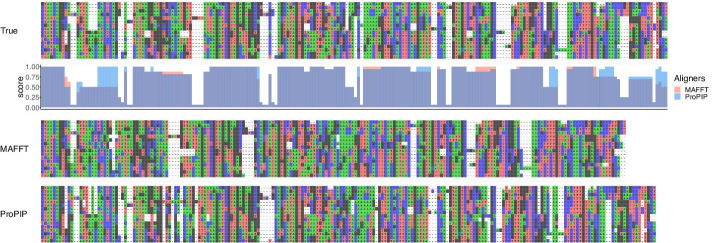
Overalignment example block #2. The 3 MSAs represent columns 1001–1188, 933–1108, and 985–1169 for true, MAFFT, and ProPIP, respectively. Analgously to Fig. 2, MAFFT produces a too short alignment while ProPIP is closer to the true MSA on top. The similarity value (AlignStat v1.3.1 [19]) is for MAFFT $81\%$ while ProPIP $86\%$ [27]. Column by column similarity values are shown under true MSA for both aligners. See also Fig. 2

Our previously published results [7] show that ProPIP does not over-align. Here we add additional experiments to illustrate this with the “distant” data from [3]. Specifically, it was generated with MySSP v.1 [16] by evolving a sequence of 1000 nucleotides under JC69 model [17] on a symmetrical 16-taxon tree with equal branch lengths of 0.075 expected substitutions/site and with the constraint of a maximum pairwise distances 0.6. The indel sizes were Poisson-distributed with averages of 1.7 bases. The synthesised dataset was aligned with both MAFFT [18] (default settings) and ProPIP, using the true tree as a guide tree. In ProPIP the indel rates have been inferred from the non-aligned sequences (as described above in paragraph Initial tree and indel rate inference). Compared to the true MSA consisting of 1213 columns, MAFFT (with option ep 0.0 to allow longer indels) inferred an MSA with 1146 columns, while ProPIP inferred a longer MSA with 1193 columns. Figs. 2, 3 show the comparison of the three MSAs focusing on two homologous blocks—the first at the beginning of the MSA and the second at its end. Quality scores have been calculated with the AlignStat [19] and Q-score [20, 21]. It can be observed that MAFFT over-aligns sequences while ProPIP generates alignments with a gap pattern more compatible with the true MSA. It is worth noting, however, that for both aligners this is a relatively complicated case where the sequences are distant from each other. By adjusting indel-related parameters (i.e., gap penalties or indel rates) of the alignment program, one can potentially change the inferred gap pattern. Nevertheless, even with indel rates that deviate from optimal values, ProPIP does not overalign and produces high quality MSA. In order to demonstrate the robustness of ProPIP to changes in insertion $\lambda$ and deletion $\mu$ rates we re-aligned the synthetic dataset 50 times, by introducing an increasing noise level $p_i$ from $1\%$ to $50\%$ to the input indel rate values. For each $p_i$ we have generated 50 uniform random samples of pairs of $\{\lambda ,\mu \}$ within the region $\left[ \lambda -\lambda \cdot p_i, \lambda +\lambda \cdot p_i,\right]$ and $\left[ \mu -\mu \cdot p_i, \mu +\mu \cdot p_i,\right]$, respectively.

**Fig. 4:**
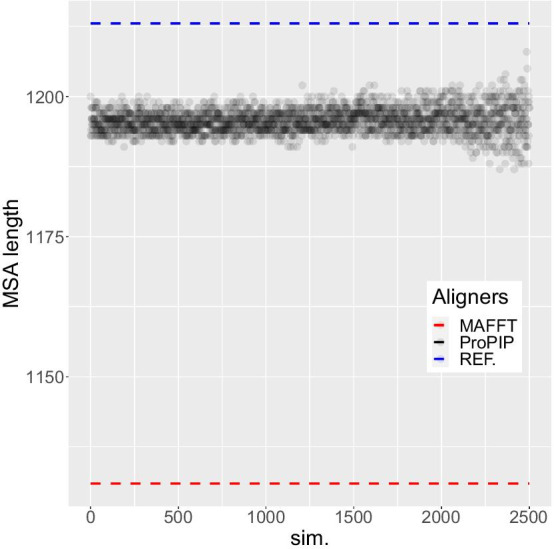
MSA length. The length of the 2500 MSAs obtained by increasingly perturbing the indel rates is represented by the black dots. The length of the reference MSAs and the length of the alignment obtained with MAFFT have also been added (dashed colored lines). ProPIP proves to be robust to a sustained disturbance of indel rates from the overalignment problem perspective

**Fig. 5:**
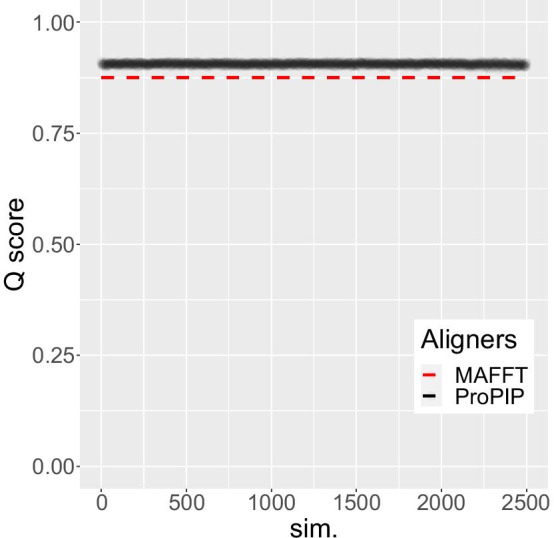
Quality score. The 2500 MSAs obtained as described in the Results section have been scored with qscore [20]. The quality scoring (Cline et al. shift score [21]) is represented with black dots while as a reference (dashed line) we have also added the score obtained by MAFFT alignment (using ep equal to 0). ProPIP proves to be very robust to a sustained disturbance of indel rates also from the MSA quality point of view

**Fig. 6:**
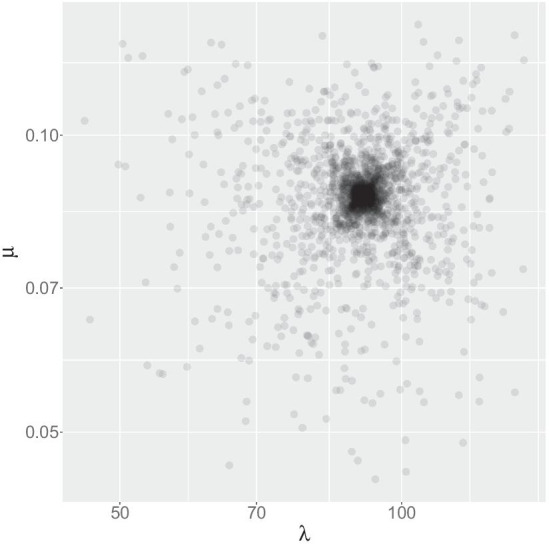
Indel rates ($\mu$ and $\lambda$). This figure represents the 2500 insertion rate and deletion rate values related to the simulations described in Results

Figures 4 and 5 show the lengths of the 2500 resulting MSAs and the relative quality scores (Cline et al. shift score [21]), the used indel rates are shown in Fig. 6. This experiment shows that compared to MAFFT, our aligner always infers MSAs that are longer and closer to the true MSA length and of a better quality, despite large deviations in input indel rates.

## Discussion and conclusion

Popular state-of-the-art alignment software typically relies on gap penalties and modifying them visibly affects the inferred gap patterns. How to set these appropriately for a given dataset is unclear. The usual practice is using default values, which are inferred by software developers empirically, for example based on real data benchmarks. However, benchmarking and tuning MSA inference is known to be notoriously difficult [22]. Default gap penalties may not be appropriate for individual datasets [23]. In addition, they are generally not phylogenetically interpretable.

In contrast, gap patterns inferred by ProPIP are controlled by insertion and deletion events that are mathematically described by a generative evolutionary process. Therefore, gap patterns inferred by ProPIP are phylogenetically consistent and require no a priori chosen gap costs. Instead, the initial indel rates are computed from the input sequences at hand and eventually can be optimised (for example by maximum likelihood). The resulting indel rates and events are biologically meaningful and, moreover, are dataset specific, *i.e*., they allow to accommodate any special features of an individual dataset, rather than relying on generic indel values.

The popular aligner PRANK was the first to correct for over-alignment using an algorithmic approach to distinguish insertions from deletions. MAFFT followed up with a different approach, based on using variable scoring matrix for different pairs or groups of sequences. ProPIP, the software presented here, avoids the over-alignment problem by using the explicit model to describe indel evolution over time.

Previously, we showed that both PRANK and our PIP-based methods produce high quality and phylogenetically consistent alignments of similar length, but vary in the inferred gap pattern [7]. The availability of an explicit model of indel evolution makes ProPIP a useful tool for systematic statistical inferences regarding the indel rates and events history.

On the other hand, one may rightfully doubt whether an MSA inference method based on a single-residue indel model like PIP is capable of inferring long gaps. While this will be the subject of a separate large-scale systematic study, our preliminary results show that ProPIP does infer long gaps when these are suggested by the data [24].

Through the phylogeny-aware explicit description of indel evolution, the PIP model leads to more plausible MSAs than more traditional methods relying on arbitrary gap penalties. ProPIP avoids overalignment, estimate indel rates, and infers gap patterns that are consistent with the phylogeny. Overall, this leads to results that have a proper biological interpretation. Note that phylogenetic aligners are sensitive to the quality of the guide tree and are likely to perform rather poorly on structural benchmarks [25, 26]. For this reason, it is essential that tree inference is also performed under a robust evolutionary model. A future goal is to infer phylogenies under PIP so that the MSA and tree share a consistent model. Finally, model-based alignment methods like ProPIP facilitate future developments towards the quantification of uncertainty in inferred MSA columns and in the estimates of parameter values.

## Data Availability

The source code can be compiled on Linux, macOS and Microsoft Windows platforms. The algorithm is implemented in C++ as standalone program. The source code is freely available on GitHub repository at https://github.com/acg-team/ProPIP and distributed under the terms of the GNU GPLv3 license. Documentation regarding the use of the tool and its features as well as some sample datasets can be found online in the software GitHub repository.

## Abbreviations

DP: Dynamic programming
PIP: Poisson Indel Process
SB: Stochastic backtracking
ASRV: Across-site rate variation
MSA: Multiple sequence alignment
TBB: Intel thread building blocks library
Indel: Insertions and deletions
